# Homocysteine and Lp-PLA2 levels: Diagnostic value in coronary heart disease

**DOI:** 10.1097/MD.0000000000035982

**Published:** 2023-11-17

**Authors:** Linlin Wu, Peng Shao, Zhanyi Gao, Shan Zhang, Jiahui Ma, Jie Bai, Yuejuan Wei

**Affiliations:** a Department of Cardiology, Cangzhou Hospital of Integrated TCM-WM Hebei, Xinhua District, Cangzhou, Hebei Province, China.

**Keywords:** coronary heart disease, diagnostic efficacy, homocysteine, inflammation, lipoprotein-associated phospholipase A2, risk factors

## Abstract

Coronary heart disease (CHD) is the leading cause of mortality worldwide. Identifying effective diagnostic markers and understanding risk factors is crucial for prevention and management. This study aimed to investigate the levels of homocysteine (Hcy) and lipoprotein-associated phospholipase A2 (Lp-PLA2) in human plasma and their roles in the diagnosis and prognosis of CHD. A retrospective study was conducted on 232 patients with CHD, divided into Acute Myocardial Infarction, unstable angina pectoris, and stable angina pectoris groups, and a control group of 75 healthy adults. Blood samples were analyzed for serum Hcy and Lp-PLA2 levels using the cycling enzyme method and ELISA method, respectively. Statistical analyses were performed to evaluate the risk factors, and diagnostic efficacy was assessed using receiver operating characteristic (ROC) curves. No significant differences in age and sex were observed between the study and control groups, whereas marked disparities in risk factors such as obesity, hypertension, diabetes, and hyperlipidemia were noted. Significant differences in serum Hcy and Lp-PLA2 levels were identified among the CHD subgroups. Univariate and multivariate logistic regression analyses revealed that Hcy, Lp-PLA2, hypertension, and hyperlipidemia were significant risk factors for CHD. The combined diagnostic Area Under the Curve (AUC) for Hcy and Lp-PLA2 was found to be higher than that when using them individually. This study identified the elevation of Hcy and Lp-PLA2 levels as independent risk factors for CHD, and their conjoint analysis significantly enhanced clinical diagnostic efficacy. These findings provide valuable insights for CHD diagnosis, treatment, and prevention, highlighting the importance of these markers in CHD management.

## 1. Introduction

Coronary heart disease (CHD) is a global health dilemma that continues to escalate in terms of both the prevalence and severity of its impact.^[1]^ This multifaceted disorder stems from the insidious progression of coronary atherosclerosis, which is characterized by the deposition of fatty plaques within the coronary arteries. Such pathological accumulation leads to serious clinical manifestations, including arterial obstruction, constriction, ischemia of the heart muscle tissue, and even culminating myocardial cell necrosis. The underlying pathology and clinical spectrum of CHD involve complex interplay between genetic predispositions, metabolic dysregulation, and environmental triggers.^[2]^ Multiple risk factors, including unhealthy diet, sedentary lifestyle, chronic stress, hypertension, hyperglycemia, and hyperlipidemia, synergistically contribute to disease onset and progression.^[3]^

Currently, coronary angiography is the gold standard for the diagnosis of CHD. However, its invasive nature coupled with high costs curtails its routine application in clinical practice, particularly in low-resource settings. This limitation has spurred an urgent need to develop more accessible, noninvasive, and cost-effective diagnostic strategies.^[4,5]^ The lacuna in the early detection of CHD often translates to delayed intervention, resulting in compromised therapeutic outcomes and unfavorable prognoses. Therefore, the quest for innovative diagnostic approaches has become a pivotal aspect of contemporary cardiovascular research.^[6]^ Identifying robust, specific, and sensitive serological tests could potentially revolutionize early CHD detection, thus facilitating timely intervention and optimal patient management.^[7]^

Recent advancements have underscored Hcy and lipoprotein-associated phospholipase A2 (Lp-PLA2) as notable biomarkers for CHD diagnosis and risk assessment.^[8,9]^ These biomarkers are appealing because of their minimal susceptibility to confounding factors and potential to reflect early cardiac injury.^[8]^ Their integration into current diagnostic methods may signify a paradigm shift towards personalized, precision medicine in CHD care. Targeted utilization and understanding of Hcy and Lp-PLA2 can lead to individualized preventive measures, more precise therapeutic interventions, and improved patient outcomes.^[10]^

The role of inflammation in cardiovascular health and disease is rapidly gaining prominence. A burgeoning body of evidence posits that inflammation is not merely an aftermath but is intricately woven into the fabric of many cardiovascular pathologies. Central to this inflammatory web are certain biochemical markers that have shown significant associations with cardiovascular outcomes. Homocysteine, a sulfur-containing amino acid, has been observed to be considerably linked with inflammation. Hyperhomocysteinemia, common in the elderly population, disrupts the blood barrier function, instigating inflammatory events that amplify disease pathology.^[11]^ Furthermore, this disruption and ensuing inflammation have been associated with grave conditions like Alzheimer disease and other neurodegenerative diseases. Similarly, Lp-PLA2, predominantly produced in macrophages, has been directly associated with vascular inflammation.^[12]^ Its role is not just confined to inflammation; Lp-PLA2 also mediates macrophage migration and activation. In the context of hypertensive cardiac remodeling, Lp-PLA2’s involvement in macrophage-mediated inflammation is significant. The matrix of inflammatory connections is further complicated by diseases like type 2 diabetes mellitus, which in itself is associated with elevated markers like neuregulin-4.^[13]^ This cytokine’s link with type 2 diabetes mellitus underscores the intricate interplay of inflammation and metabolic aberrations. Likewise, the uric acid to HDL-cholesterol ratio emerges as a potential predictor of metabolic syndrome in type 2 diabetic subjects and also as a valuable marker in hypertensive patients reflecting the potential inflammatory underpinnings of these conditions.^[14,15]^ Moreover, diabetic kidney injury acts as a testament to the intertwining of diabetes, renal health, and inflammation. With elevated uric acid to HDL-cholesterol ratio levels seen in patients with diabetic kidney injury, the inflammatory pathways in diabetic microvascular complications become evident.^[15,16]^ Given this confluence of inflammatory pathways and their potential role in the onset, progression, and prognosis of cardiovascular diseases, it becomes paramount to delve deeper into these markers. Understanding homocysteine, Lp-PLA2, and other related markers in the context of inflammation and cardiovascular health can usher in an era of personalized, precise diagnostic, and therapeutic strategies in cardiovascular care.

Comprehensive research and validation are still required; however, these promising molecules pave the way for a novel and more accessible approach to CHD diagnosis and management. Given this pressing need and promising scientific landscape, the present research will embark on exhaustive exploration of Hcy and Lp-PLA2. By performing a rigorous comparative analysis of these biomarkers in the serum of patients with CHD versus healthy controls, this study sought to discern their diagnostic efficacy. Furthermore, the correlation between these biomarkers and various known and potential risk factors for CHD was assessed.

## 2. Materials and Methods

### 2.1. Study design

This retrospective study was conducted from May 2021 to May 2022 at Cangzhou Hospital of Integrated TCM-WM Hebei Hospital, focusing on the levels of Hcy and Lp-PLA2 in human plasma and their diagnostic and prognostic value in CHD. Patients were carefully selected, and 232 patients with CHD were included in the study. The patients were divided into 3 distinct groups based on the underlying condition: acute myocardial infarction (AMI) group (n = 78), unstable angina pectoris (UAP group, n = 76), and stable angina pectoris (SAP group, n = 78). The participants ranged in age from 48 to 85 years, including 135 males and 97 females. All patients were meticulously diagnosed using coronary angiography to confirm their condition. Individuals with cerebrovascular diseases, malignant tumors, mental illness, autoimmune disorders, and severe infections were excluded from the study. This ensured a more focused and homogeneous patient population. A control group consisting of 75 healthy adults (46 males and 29 females, aged 45–79 years) who underwent physical examination in the same hospital during the study period was also formed. This group served as a reference to draw valuable comparisons and insights. All participants provided informed consent, and the study protocol was approved by the Ethics Committee of the Cangzhou Hospital of Integrated TCM-WM Hebei (2021-073).

### 2.2. Sample collection and assays

Blood samples were collected from CHD patients in the morning following admission and after 12 hours of fasting for the control group. Samples were placed in yellow-capped tubes containing a clotting gel and then centrifuged at 3500 rpm for 10 minutes. The supernatant was used to measure serum concentrations of Hcy and Lp-PLA2. The serum concentration of Hcy was determined using the cycling enzyme method on a Roche MODULAR P800 Automatic Biochemistry Analyzer. For Lp-PLA2 concentration, ELISA was performed using an Addcare ELISA 1100 Automatic Enzyme Immunoassay System.

### 2.3. Statistical analysis

The data were analyzed using SPSS 22.0. Before the primary statistical analyses, the data’s normality was verified using the Shapiro–Wilk test. Once the data was confirmed to follow a normal distribution, subsequent analyses were carried out. Quantitative data are expressed as the mean ± standard deviation, and intergroup comparisons were performed using the *t*-test. Count data are presented as percentages and analyzed using the χ^2^ test. Single-factor variance analysis was conducted on indicators for the control, AMI, UAP, and SAP groups. If there were significant differences, further pairwise comparisons were made using the LSD-t test. Univariate and multivariate logistic regression analyses were used to validate the risk factors of patients with CHD. The diagnostic efficacy of serum Hcy and Lp-PLA2 levels for CHD patients with CHD was evaluated using ROC curves, with the AUC used for description and comparison.

## 3. Results

Before diving into detailed statistical analyses, the data’s distribution was assessed for normality. The Shapiro–Wilk test was utilized for this purpose. The results of the test confirmed that all data adhered to a normal distribution.

### 3.1. Comparative clinical characteristics of control and study groups

In the comparison of clinical features between the study and control groups, there were no statistically significant differences in terms of age and sex (*P* > .05). As summarized in Table 1, marked disparities were noted in the positive probability of other observation indices, including obesity, hypertension, diabetes, hyperlipidemia, long-term smoking, and long-term alcohol consumption, in the CHD group compared to the control group (*P* < .05).

**Table 1:** Comparative analysis of clinical characteristics between control and study groups [n (%)].

Group	n	Age (≥60, yrs.)	Male	Female	Obesity (BMI > 25 kg/m²)	Hypertension	Diabetes	Hyperlipidemia	Long-term smoking	Long-term alcohol consumption
Control	75	55 (73.3)	46 (57.5)	29 (36.2)	20 (25.0)	15 (18.7)	13 (16.2)	14 (17.5)	24 (30.0)	22 (27.5)
Study	232	178 (76.7)	135 (55.6)	97 (39.9)	95 (39.1)	123 (50.6)	77 (31.7)	144 (59.2)	108 (44.4)	97 (39.9)
*χ*² value	–	0.520	0.035	0.031	5.134	26.009	9.122	43.640	5.138	7.375
*P* value	–	0.285	0.480	0.482	0.016	0.001	0.002	0.000	0.017	0.005

### 3.2. Comparison of serum indicators among different coronary heart disease subgroups

When comparing the levels of serum Hcy and Lp-PLA2 among the control, SAP, UAP, and AMI groups, significant differences were observed (*P* < .01). These differences in serum markers highlight variations in biochemical characteristics and can be instrumental in understanding different CHD subtypes. The results are presented in Table 2.

**Table 2:** Comparison of serum indicators among different CHD subtypes.

Group	n	Hcy (μmol/L)	Lp-PLA2 (ng/mL)
Control	75	8.23 ± 1.97	84.25 ± 32.18
SAP group	78	11.81 ± 2.07	123.56 ± 28.78
UAP group	76	15.14 ± 3.60	152.31 ± 54.90
AMI group	78	21.48 ± 9.82	184.12 ± 58.52
*F*/*P* value	–	23.74/0.000	19.04/0.000

AMI = acute myocardial infarction, SAP = stable angina pectoris, UAP = unstable angina pectoris.

### 3.3. Analysis of univariate factors influencing coronary heart disease in patients

A univariate logistic regression analysis using the backward elimination method was conducted to elucidate the role of several risk factors in CHD. The backward elimination approach was chosen to start with a full model and iteratively remove the least significant predictors, ensuring that the final model only contains variables that contribute meaningfully to the prediction of CHD.

A univariate logistic regression analysis was conducted to elucidate the role of several risk factors in CHD (Table 3). The analysis revealed that certain factors, namely, Hcy, Lp-PLA2, hypertension, diabetes, and hyperlipidemia, were significantly associated with CHD. The OR values indicate the odds ratio for each factor, demonstrating a particular influence on CHD occurrence. Interestingly, long-term smoking and drinking did not show significant statistical associations, in contrast to some existing studies. However, the results related to Hcy and Lp-PLA2 were consistent with prior research, reaffirming their role in CHD pathogenesis. The findings of this study could be integral to designing targeted interventions for CHD prevention and treatment, taking into account individual risk profiles.

**Table 3:** Univariate logistic regression analysis of CHD patients.

Indicator	OR value	95% CI	*P* value
Hcy	0.400	1.380–13.495	.027
Lp-PLA2	1.050	1.020–1.080	.011
Obesity	0.550	0.152–1.999	.305
Hypertension	0.254	0.066–0.977	.036
Diabetes	0.397	0.035–0.618	.050
Hyperlipidemia	0.184	0.047–0.712	.012
Long-term Smoking	2.369	0.688–8.163	.232
Long-term Drinking	0.466	0.130–1.679	.210

Hcy = homocysteine, Lp-PLA2 = lipoprotein-associated phospholipase A2.

### 3.4. Comprehensive examination of multifactorial influences on coronary heart disease

A stepwise multivariate logistic regression analysis was employed to provide a more intricate understanding of the risk factors contributing to CHD. The stepwise method was chosen to iteratively select the most significant predictors while eliminating those that do not contribute meaningfully to the model. This approach allows for a comprehensive exploration of the interplay between various independent variables and their collective influence on the outcome.

Multivariate logistic regression analysis provided a more complex understanding of the risk factors contributing to CHD (Table 4). This statistical method allows the exploration of the interplay between various independent variables and how they might interact to influence the outcome. Notably, this analysis underscored the significant association between CHD and 4 particular factors: Hcy, Lp-PLA2, hypertension, and hyperlipidemia. Each of these factors demonstrated a substantial relationship with CHD, and their respective OR elucidated the likelihood of CHD occurrence in the presence of these conditions. Specifically, the observed increase in the OR for Hcy and Lp-PLA2 and the decrease in the OR for hypertension and hyperlipidemia highlight the potential multifactorial nature of CHD.

**Table 4:** Multivariate logistic regression analysis of CHD patients.

Indicator	OR value	95% CI	*P* value
Hcy	1.376	1.280–14.863	.040
Lp-PLA2	1.014	1.024–1.081	.012
Hypertension	0.221	0.099–0.977	.038
Hyperlipidemia	0.140	0.036–0.742	.015

Hcy = homocysteine, Lp-PLA2 = lipoprotein-associated phospholipase A2.

### 3.5. Diagnostic efficacy of serum homocysteine and lipoprotein-associated phospholipase A2 in coronary heart disease

**Figure 1.**
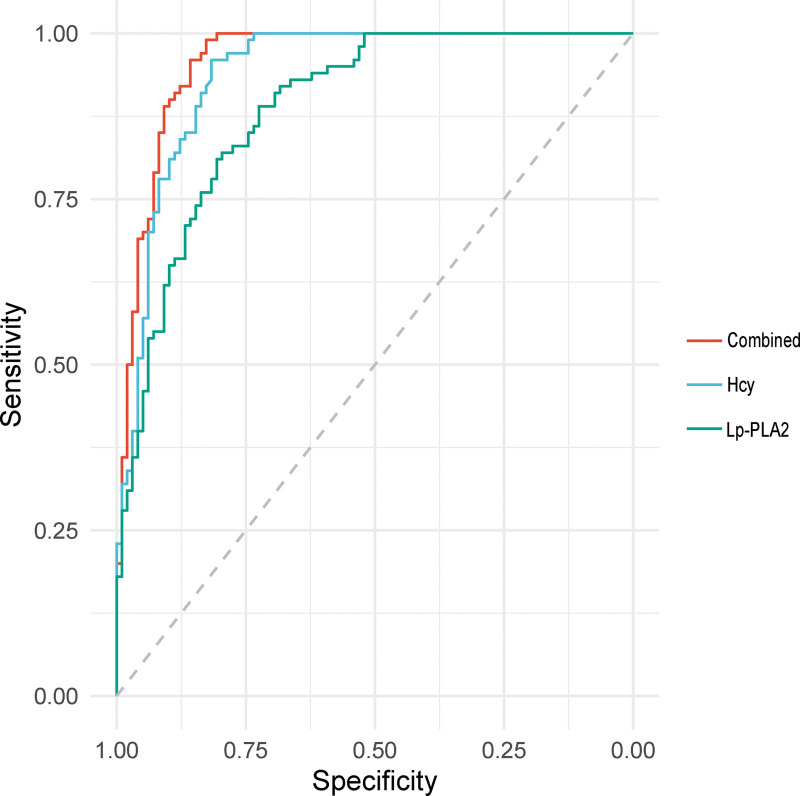
Diagnostic efficacy of Hcy, Lp-PLA2, and combined testing for coronary heart disease. Hcy = homocysteine, Lp-PLA2 = lipoprotein-associated phospholipase A2.

By employing ROC curves to compare the diagnosis of CHD using serum Hcy, Lp-PLA2, and their combination, the results showed that the AUC for the combined indices was 0.957, which was distinctly higher than that of Hcy and Lp-PLA2 alone (0.939 and 0.889, respectively). This underscores the enhanced sensitivity of combined testing and signifies a remarkable increase in diagnostic effectiveness, thereby suggesting an optimized approach for CHD screening and management. Figure 1 presents a visual representation of this finding.

## 4. Discussion

CHD is a prevalent cardiovascular disorder with a multifactorial etiology that is commonly linked to genetic predispositions, environmental factors, and lifestyle habits.^[4,5]^ Despite significant advances in our understanding of CHD prevention and treatment, it continues to be recognized as one of the principal causes of mortality in humans. CHD complexity arises from the interaction of various risk factors, including the degree of inflammation, thinning of the fibrous cap, metalloproteinase expression levels, plaque characteristics, and stability.^[6]^ As the disease progresses, if not diagnosed and promptly prevented, complications, disability rates, and mortality are notably escalated. Thus, there is an urgent clinical need to identify a noninvasive detection index that would alleviate the discomfort of angiography, reduce medical expenses, and provide early identification of CHD. Traditional clinical approaches for the early detection of CHD rely on serum cholesterol and CRP markers. However, studies have found that their predictive value is limited, as most patients with total cholesterol levels are comparable to those without cardiovascular diseases.^[17]^ CRP, an acute-phase reactant, lacks specificity, as it can increase under various conditions.

Hcy, a thiol-containing amino acid, is an intermediate product of methionine and cysteine metabolism. The human body does not synthesize it and it can only be obtained through dietary intake. Abnormal metabolism may lead to Hcy accumulation, with high levels damaging endothelial cells, inducing vascular smooth muscle cell proliferation, and accelerating collagen deposition, leading to overreactive inflammatory responses and thrombus formation, thus obstructing lipid metabolism, inducing endothelial dysfunction, and atherosclerosis, ultimately leading to thrombosis.^[18,19]^ The current research indicates that higher serum levels of Hcy correlate with less stable and more constricted coronary atherosclerotic plaques, providing essential evidence for early diagnosis in patients with CHD.

Lp-PLA2, an enzyme produced by macrophages and monocytes, plays a vital role in proinflammatory phospholipid metabolism and atherosclerotic metabolites.^[20]^ Enzyme activity in unstable plaques was found to be significantly higher than that in stable plaques.^[21]^ This highlights the significant correlation between Lp-PLA2 activity and plaque stability, making it a reliable and valuable indicator for identifying vulnerable plaques and assessing cardiovascular risks. The study revealed higher levels of Lp-PLA2 in less stable coronary artery plaques, possibly due to harmful substances, such as Lyso-PC and oxNEFAs, that are broken down by Lp-PLA2. They can recruit leukocytes, upregulate inflammatory cytokines, and enhance oxidation and metalloproteinase expression, leading to an expansion of the necrotic lipid core and thinning of the fibrous cap, resulting in plaque rupture.

Using logistic regression analysis, this study identified CHD risk factors, such as Hcy, Lp-PLA2, hypertension, diabetes, and hyperlipidemia, while obesity, chronic alcohol consumption, and smoking were found to be unrelated. Moreover, 50% to 70% of CHD patients have concomitant hypertension, leading to arteriosclerosis and stenosis if not effectively controlled. Hyperlipidemia remains the most significant risk factor, causing blood thickening, atherosclerosis formation, and eventually, CHD.^[6]^ Thus, besides unmodifiable factors such as sex, age, and genetics, optimizing lifestyle and dietary habits to reduce controllable risks is pivotal in preventing CHD. The research showed that Hcy and Lp-PLA2 have higher diagnostic values for patients with CHD. Through ROC curve analysis, the combined testing of serum Hcy and Lp-PLA2 levels in patients with CHD demonstrated higher diagnostic efficacy than single indicators. This signifies the potential of these biomarkers as innovative tools for the early detection and management of CHD, paving the way for personalized treatment strategies and patient care.

Given the escalating prevalence of CHD globally, our research presents implications for its clinical management. The pronounced disparities in Hcy and Lp-PLA2 levels among CHD subgroups, as delineated in our findings, illuminate potential avenues for patient stratification and targeted therapeutic interventions. Specifically, the diagnostic efficacy of combined analysis of serum Hcy and Lp-PLA2 levels underscores their potential use as concurrent biomarkers, amplifying the precision in CHD detection. Our study’s emphasis on the significant associations between Hcy, Lp-PLA2, hypertension, and hyperlipidemia with CHD suggests a roadmap for a multifaceted approach to CHD management. The identification of these risk factors not only aids in the prediction of CHD risk but can also guide interventions, promoting primary prevention in high-risk populations. This could facilitate early detection, timely intervention, and potentially improved patient outcomes. Moreover, the stepwise multivariate logistic regression analysis, which illuminated the multifactorial nature of CHD, emphasizes the need for an integrated care model. Addressing CHD would require tackling not just the primary cardiac abnormalities but also the underlying conditions like hypertension and hyperlipidemia, which contribute to the disease’s pathogenesis. Furthermore, the utility of the ROC curves in our study presents a promising approach for clinical settings, facilitating the evaluation of the predictive accuracy of diagnostic tests. The superior AUC value for combined indices compared to individual biomarkers highlights the robustness of combining multiple biomarkers, pointing towards a more comprehensive diagnostic strategy. In conclusion, our study’s findings pave the way for advancing personalized medicine in cardiovascular care. By pinpointing key risk factors and enhancing diagnostic accuracy, we hope to optimize therapeutic interventions, improving patient outcomes and potentially reducing the healthcare burden associated with CHD.

This study had several limitations that must be acknowledged. The sample size may not reflect a broader population, potentially limiting the generalizability of the findings. Additionally, the cross-sectional nature of the research design may not fully capture the complex dynamics of homocysteine and lipoprotein-associated phospholipase A2 in coronary heart disease progression. An important limitation to note is that while our study extensively investigated the roles of Hcy and Lp-PLA2, it did not delve into other potentially significant biomarkers, such as NT-proBNP. As noted, NT-proBNP, renowned for its association with cardiac stress, could offer additional insights into the disease’s diagnosis and prognosis. Future studies incorporating a more diverse array of biomarkers can provide a holistic understanding of the intricate interplay between various biomarkers and their collective influence on CHD. Future research with a longitudinal design and diverse populations could offer more comprehensive insight into these relationships.

## 5. Conclusions

In conclusion, the elevation of Hcy and Lp-PLA2 levels in the serum of patients with CHD was distinctly identified in this study. These elements, along with hypertension and hyperlipidemia, are independent risk factors for CHD and provide an invaluable basis for its diagnosis, treatment, and prevention. Conjoint analysis of Hcy and Lp-PLA2 significantly enhanced the clinical diagnostic efficacy for CHD patients, providing a novel approach for clinical diagnosis and treatment.

## Acknowledgments

We appreciate the cooperation and informed consent provided by the patients in this study.

## Author contributions

**Data curation:** Peng Shao.

**Formal analysis:** Zhanyi Gao.

**Investigation:** Linlin Wu.

**Methodology:** Shan Zhang.

**Resources:** Jiahui Ma, Jie Bai.

**Software:** Jiahui Ma.

**Supervision:** Yuejuan Wei.

**Visualization:** Jie Bai.

**Writing – original draft:** Linlin Wu.

**Writing – review & editing:** Yuejuan Wei.

## References

[1] Dalen JE Alpert JS Goldberg RJ. The epidemic of the 20(th) century: coronary heart disease. Am J Med. 2014;127:807–12. 24811552 10.1016/j.amjmed.2014.04.015

[2] Wirtz PH von Känel R. Psychological stress, inflammation, and coronary heart disease. Curr Cardiol Rep. 2017;19:111. 28932967 10.1007/s11886-017-0919-x

[3] Liu W Wang T Sun P. Expression of Hcy and blood lipid levels in serum of CHD patients and analysis of risk factors for CHD. Exp Ther Med 2019;17:1756–60. 30783445 10.3892/etm.2018.7111 PMC6364198

[4] Hoffmann U Ferencik M Cury RC. Coronary CT angiography. J Nucl Med. 2006;47:797–806. 16644750

[5] Pöhler E Günther H Diekmann M. Outpatient coronary angiography--safety and feasibility. Cardiology. 1994;84:305–9. 8187117 10.1159/000176416

[6] Karagiannidis E Moysidis DV Papazoglou AS. Prognostic significance of metabolomic biomarkers in patients with diabetes mellitus and coronary artery disease. Cardiovasc Diabetol. 2022;21:70. 35525960 10.1186/s12933-022-01494-9 PMC9077877

[7] Drakopoulou M Toutouzas K Stefanadi E. Association of inflammatory markers with angiographic severity and extent of coronary artery disease. Atherosclerosis. 2009;206:335–9. 19264307 10.1016/j.atherosclerosis.2009.01.041

[8] Schaffer A Verdoia M Cassetti E. Relationship between homocysteine and coronary artery disease Results from a large prospective cohort study. Thromb Res. 2014;134:288–93. 24928335 10.1016/j.thromres.2014.05.025

[9] Sofogianni A Alkagiet S Tziomalos K. Lipoprotein-associated phospholipase A2 and coronary heart disease. Curr Pharm Des. 2018;24:291–6. 29332572 10.2174/1381612824666180111110550

[10] Dohi T Miyauchi K Ohkawa R. Higher lipoprotein-associated phospholipase A2 levels are associated with coronary atherosclerosis documented by coronary angiography. Ann Clin Biochem. 2012;49(Pt 6):527–33. 22933444 10.1258/acb.2012.011252

[11] Tawfik A Elsherbiny NM Zaidi Y. Homocysteine and age-related central nervous system diseases: role of inflammation. Int J Mol Sci . 2021;22:6259. 34200792 10.3390/ijms22126259 PMC8230490

[12] S-l Lv Zeng Z-f Gan W-q. Lp-PLA2 inhibition prevents Ang II-induced cardiac inflammation and fibrosis by blocking macrophage NLRP3 inflammasome activation. Acta Pharmacol Sin. 2021;42:2016–32. 34226664 10.1038/s41401-021-00703-7 PMC8632984

[13] Sincer I Gunes Y Mansiroglu AK. Association of mean platelet volume and red blood cell distribution width with coronary collateral development in stable coronary artery disease. Postepy Kardiol Interwencyjnej 2018;14:263–9. 30302102 10.5114/aic.2018.78329 PMC6173096

[14] Kocak MZ Aktas G Erkus E. Neuregulin-4 is associated with plasma glucose and increased risk of type 2 diabetes mellitus. Swiss Med Wkly. 2019;149:w20139. 31656034 10.4414/smw.2019.20139

[15] Kocak MZ Aktas G Erkus E. Serum uric acid to HDL-cholesterol ratio is a strong predictor of metabolic syndrome in type 2 diabetes mellitus. Rev Assoc Med Bras (1992) 2019;65:9–15. 30758414 10.1590/1806-9282.65.1.9

[16] Aktas G Yilmaz S Kantarci DB. Is serum uric acid-to-HDL cholesterol ratio elevation associated with diabetic kidney injury? Postgrad Med. 2023;135:519–23. 37170820 10.1080/00325481.2023.2214058

[17] Moretti R Caruso P. The controversial role of homocysteine in neurology: from labs to clinical practice. Int J Mol Sci . 2019;20:231. 30626145 10.3390/ijms20010231 PMC6337226

[18] Mangoni AA Woodman RJ. Homocysteine and cardiovascular risk an old foe creeps back. J Am Coll Cardiol. 2011;58:1034–5. 21867838 10.1016/j.jacc.2011.05.029

[19] Knuuti J Wijns W Saraste A. 2019 ESC guidelines for the diagnosis and management of chronic coronary syndromes. Eur Heart J. 2020;41:407–77. 31504439 10.1093/eurheartj/ehz425

[20] Huang F Wang K Shen J. Lipoprotein-associated phospholipase A2: the story continues. Med Res Rev. 2020;40:79–134. 31140638 10.1002/med.21597 PMC6973114

[21] Maiolino G Bisogni V Rossitto G. Lipoprotein-associated phospholipase A2 prognostic role in atherosclerotic complications. World J Cardiol 2015;7:609–20. 26516415 10.4330/wjc.v7.i10.609 PMC4620072

